# Intimate partner violence and its associated factors among pregnant women in Bale Zone, Southeast Ethiopia: A cross-sectional study

**DOI:** 10.1371/journal.pone.0214962

**Published:** 2019-05-01

**Authors:** Bikila Lencha, Gemechu Ameya, Girma Baresa, Zanebe Minda, Gemechu Ganfure

**Affiliations:** 1 Department of Public Health, Goba Referral Hospital, Madda Walabu University, Bale-Goba, Oromia, Ethiopia; 2 Department of Medical Laboratory Science, College of Medicine and Health Sciences, Arba Minch University, Arba Minch, Ethiopia; 3 Department of Nursing, Goba Referral Hospital, Madda Walabu University, Bale-Goba, Oromia, Ethiopia; 4 Department of Midwifery, Goba Referral Hospital Madda Walabu University, Bale-Goba, Oromia, Ethiopia; Aga Khan University, KENYA

## Abstract

**Background:**

Intimate partner violence (IPV) against women is a major public health concern in low income countries. Violence against pregnant women has adverse effects on maternal and newborn outcomes. This study aimed to assess the prevalence and associated factors of intimate partner violence in Southeast Ethiopia pregnant women.

**Methods:**

Institutional based cross-sectional study was conducted on pregnant women who were attending antenatal care (ANC) in Bale Zone health institution during study period. Face to face interviews were conducted using a pre-tested structured questionnaire. Data related to socio-demographic characteristic, pregnancy and reproductive history, intimate partner behavior and IPV encountered during recent pregnancy was gathered for this study. Descriptive analysis and logistic regression were used for the data analysis. Odds ratio with 95% CI was computed to determine the presence and strength of associated factors with IPV.

**Results:**

A total of 612 pregnant women participated in the study. Of these, 361 (59.0%) pregnant women faced at least one type of IPV during the recent pregnancy. Physical violence (20.3%), sexual violence (36.3%), psychological/emotional violence (33.0), controlling behavior violence (30.4%) and economic violence (27.0) were the type of IPV encountered by participants. An intimate partners who were drank alcohol [AOR = 2.9; 95% CI: (1.5–5.4)], partners who were chewed Khat [AOR = 1.7; 95% CI: (1.1–2.6)], partners who were smoked cigarette [AOR = 2.6; 95% CI: (1.4–4.9)], partners who had aggressive behavior [AOR = 2.8; 95% CI: (1.7–4.6)], having partner age ≥30 year old [AOR = 1.8; 95% CI: (1.2–2.9)], unwanted pregnancy [AOR = 3.3; 95% CI: (1.9–5.5)] and history of adverse pregnancy outcome [AOR = 2.1; 95% CI: (1.2–3.6)] that were the factors that significantly associated with IPV of the pregnant women.

**Conclusion:**

The prevalence of IPV during pregnancy was high among the study participants. Intimate partners’ use of substance, intimate partners’ aggressive behavior, older intimate partners, unwanted pregnancy and history of adverse birth outcome were identified as associated factors for IPV. IPV needs to be considered during ANC service and integrated into the sexual and reproductive health education. Community-based interventions should be advocated as a way of health promotion. Counseling, awareness creation, service provision and program design on IPV is mandatory to minimize the victim.

## Introduction

Intimate partner violence (IPV) refers to any behavior within an intimate relationship that causes physical, psychological, sexual, controlling and economic violence against a partner in the relationship [1]. Intimate partner is the husband/companion with whom the woman is having sexual relationships or the father of the child that she is carrying in her womb. IPV affects pregnant women’s health and sometimes results in maternal mortality and adverse newborn out come. Maternal distress, inadequate prenatal care, preterm birth, low birth weight, miscarriage, induced abortion, spontaneous abortion, intra uterine growth restriction, pre-eclampsia, third trimester bleeding, premature rupture of membranes, sexually transmitted infections and maternal death are associated with intimate partner violence during pregnancy [2–5]. This effect is often not recognized by policy-makers and local stakeholders working on women’s health [6–8].

High rates of IPV and maternal mortality ratios in low income countries are recognized as a global public health problem. There is a link between maternal mortality and domestic violence during or at the end of pregnancy [8, 9]. However, the problem is neglected in low income countries even though all proclaim that without addressing violence against women it is difficult to achieve expected growth and development targets [10].

Life time domestic violence against women by a husband or intimate partner in Ethiopia ranged from 19.2–78.0% [8, 9, 11, 12]. Study showed that in some area up to75% of the women believe that it is acceptable for a man to beat his wife [9]. This perception perpetuates the violence against women and needs to be changed. More than one-third of ever-married women in the country reported that they have experienced physical, emotional, or sexual violence from their husband or partner at some point in time [11]. The study suggests that pregnant women may be particularly at risk of IPV from their male partners [13].

Different factors may associate with IPV against pregnant women. Reproductive history, women related factors, intimate partner related factors, and family related factors can affect the type and magnitude of IPV against pregnant women [1, 8, 9, 13, 14]. Identifying these factors in a given society is the critical step to minimize the frequency of IPV and adverse outcome on maternal and child health. A systematic review of intimate partner violence against pregnant women in Africa showed that there is significant association between HIV infection and IPV during pregnancy [15]. Some underlying causes are well-known factor for IPV. For instance, alcohol abuse of male intimate partner had a strong association with IPV of pregnant women [15].

Although the consequence of intimate partner violence during pregnancy is worst, studies conducted so far were mostly focused on IPV of non-pregnant women. The magnitude of IPV during pregnancy and its associated factors were not well addressed in sub-Saharan countries including Ethiopia [12]. Assessing this problem among pregnant women helps government officials, policy makers, program designers and non-governmental organizations to design prevention and controlling strategies. Therefore, the aim of this study was to assess the prevalence of IPV and associated factors among Bale Zone pregnant women, Southeast Ethiopia.

## Methods

### Study design and setting

An institutional based cross-sectional study design was used to study IPV on randomly selected pregnant women who were attending in Bale zone public hospitals during the study period. Bale zone of the Oromia regional state is located in southeast of Ethiopia in the distance of 430 km from Addis Ababa (capital city of Ethiopia). There are 4 Hospitals (Goba Referral Hospital, Robe, Dalomana and Ginnir) and 87 Health Centers in the Zone. There were about 62,713 pregnant women in the study area during the period of data collection.

### Study population

The study population was drawn from pregnant women who were attending ANC clinic in Bale Zone health facilities. The study population was randomly selected after considering the inclusion and exclusion criteria. Those women who were in labor and who do not live with their intimate partner were excluded from the study.

### Sample size calculation and sampling procedure

The sample size was calculated by a single population proportion formula using a prevalence of 44.5% [12], 95% confidence interval, 5% degree of precision and a 10% possible non-response, resulting in 417 participants. On adjusting for a 1.5 design effect of multistage sampling, the final sample size was estimated to be 626.

Four districts namely Madda Walabu, Dalomana, Goro and Dinsho were randomly selected by lottery method from all districts found in the Bale zone. The estimated number of pregnant women living in each district was obtained from the zonal health office. Based on the obtained information, the sample size for each district was proportionally allocated. The health institutions found in the selected district were randomly selected. Finally, the study subjects were proportionally allocated for each selected institutions.

The pregnant women in the selected health institution were selected by systematic random sampling technique. The previous average number of pregnant women attending ANC clinic of each selected health institute in the similar period were taken from registration book and used to determine the sampling interval. The sampling interval was calculated by dividing the estimated pregnant women attending in each health institution in the study period for allocated sample size for the health institution. Finally the study subject participated in the study was selected by every K^th^ number of pregnant women coming to the health institute for ANC service.

### Study variables

The mothers were asked if they faced any act of violence during recent pregnancy. The main outcome variable in the study was intimate partner violence (women who experience any act of physical, sexual, psychological, economic violence and controlling behavior violence) during the current pregnancy. Other variables such as socio-demographic and socio-economic characteristics, intimate partner related factors, family related factors, pregnancy and reproductive history of the participants and other confounders were collected.

### Data collection tool and procedure

Face to face interviews were conducted using a pre-tested structured questionnaire which was adapted from WHO policy guidelines [6]. The modification was done to address the potential confounders in associated factors of IPV of pregnant women after reviewing the similar study conducted in different areas [2, 3, 8, 10, 12, 14]. The questionnaire was prepared in English then translated to local language (Afaan Oromo) and translated back to English to ensure consistency and accuracy. The tool contains four sections with multiple questions in each section. The questions in the data collection tool related to socio-demographic characteristics of respondents and their partner, pregnancy and reproductive history, history of physical, sexual, psychological, economic violence and controlling behavior.

The questionnaire was pretested on 5% of the sample prior to the actual data collection time on non-selected health institutions and the questionnaire was revised for possible modification. Internal consistence of the questions used to assess for IPV by using SPSS and the overall Cronbach’s alpha was 0.89. In addition to this, the content validity of the questionnaire was checked by using content validity index method. Female data collectors and supervisors were recruited as per the WHO ethical guideline [16] based on previous experience on data collection and fluency in the local languages. We gave training for one day on how to interview, handling ethical issues and maintaining confidentiality and privacy.

### Data processing and analysis

Data was first checked manually for completeness then coded and entered into Epi Info version 7.0 and exported to SPSS version 21.0 for analysis. Exploratory data analysis was done to check missing values and outliers. Bivariate logistic regression analysis was used to see the association between each of the independent variables and IPV. Thereafter, independent variables with P ≤ 0.20 in univariate logistic regression analysis was transferred to multivariable logistic regression model together, and analyzed using backward stepwise logistic regression analysis. Hosmer and Lemeshow goodness of fit of the model was used and the value was 0.47. The overall percentage explained by the model was 76.8%. The statistical significance was declared at P value less than 0.05. The results were reported using adjusted odds ratio (AOR) with their 95% confidence interval.

### Ethical consideration

Ethical clearance and approval was obtained from Ethical Review committee of Madda Walabu University. Letter of permission was obtained from each district administration and health institution. After the purpose of the study was explained, a written and verbal consent were obtained from respondents before the data collection. The interview with the pregnant women was conducted privately in the separate room. The data collectors were female health workers who were also trained to assist those participants for whom the interview could trigger an emotional response that warranted counseling. The WHO ethical and safety recommendation protocol for research on domestic violence against women was followed [16].

## Results

### Socio-demographic and reproductive history of the participants

Six hundred and twelve pregnant women participated in the study with the response rate of 97.8%. The mean age and standard deviation of the pregnant women was 26.3 ± 5.8 years and the age range was from 15–45 years old. About two-thirds of the study participants’ age ranged from 20–34 year old while the teenage is one word accounted for 23% of the study participants. All the pregnant women who participated in the study were married. Slightly less than two third of the participants were housewives. About 44.6% of participants were illiterate (unable to read and write) and 62.6% of the respondents were living in rural areas. Decision on household issues was made by both intimate partners in the majority of participants. More than three fourth of the respondents had given birth before the current pregnancy and slightly more than a quarter of the respondents didn’t want the recent pregnancy. One hundred and twelve (18.3%) of the participants had a history of pregnancy that was miscarried, aborted or ended in still birth (**Table 1**).

**Table 1:** Socio-demographic characteristics of the pregnant women participated in the study.

Variable	Frequency	Percent
**Age**		
15–19	141	23.0
20–34	401	65.6
35–45	70	11.4
**Religion**		
Muslim	488	79.7
Orthodox	106	17.3
Protestant	18	2.9
**Occupation**		
Housewife	387	63.2
Farmer	105	17.2
Government employee	52	8.5
Others*	68	11.1
**Educational status**		
Unable to read and write	273	44.6
Able to read and write without formal education	80	13.1
Primary (Grade 1–8)	158	25.8
Secondary and above	101	16.5
**With whom do you live?**		
With my husband	519	84.8
With my husband family	57	9.3
With my family of birth	29	4.7
Others**	7	1.1
**Family size**		
≤ 4	345	56.4
≥5	267	43.6
**Decision making power**		
Husband	220	35.9
Wife	12	2.0
Together	373	60.9
Other***	7	1.1

*Student, merchant

**live separately

***together with total family

### Socio-demographic and behavioral characteristics of the participants’ intimate partner

The mean age and standard deviation of the pregnant women’s intimate partners was 36.4 ± 9.8 years and the age range was from 18–75 years. More than a quarter of the respondents’ partners were illiterate (unable to read and write). Slightly more than two third of intimate partners of the women were farmers followed by merchants 15.2%. About two thirds of the intimate partners had substance usage behavior with the specific frequency of 36%, 18% and 12% for Khat chewing, cigarette smoking and alcohol drinking habit, respectively (**Table 2**).

**Table 2:** Socio-demographic characteristics of intimate partner of the participants.

Variable	Frequency	Percent
**Age**		
18–29	134	21.9
≥30	478	78.1
**Educational status**		
Unable to read and write	168	27.5
Able to read and write	133	21.7
Primary(1–8)	159	26.0
Secondary and above	152	24.8
**Occupation**		
Farmer	414	67.6
Merchant	93	15.2
Government employee	83	13.6
Others*	22	3.6
**Husband alcohol drinking habit**		
No	514	84.0
Yes	98	16.0
**Husband Khat chewing habit**		
No	379	61.9
Yes	233	38.1
**Husband cigarette smoking habit**		
No	475	77.6
Yes	137	22.4

*Student, unemployed, driver

### Prevalence of intimate partner violence

About six in ten pregnant women (59%) faced at least one form of intimate partner violence during current pregnancy. Sexual violence was the most prevalent 222 (36.3%) form of IPV encountered by pregnant women during the current pregnancy. The most frequent (31%) victim in sexual violence was forced to have sexual intercourse due to fear of intimate partner whereas the less frequent was forcing to do something sexual that is degrading or humiliating the participant. One hundred twenty four (20.3%) women experienced physical violence by their husband during the current pregnancy. Among the type of physical violence slapping or throwing something to the pregnant women was the most frequent while threatening to use or actually use a gun, knife or other weapon was less frequent 13(2.1%). One third of the respondents experienced psychological violence. Majority of the respondents were insulted or made to feel bad about themselves, 154 (25.2%). The prevalence of controlling behavior and economic violence during pregnancy was 186 (30.4%) and 165 (27%), respectively (**Table 3**).

**Table 3:** Intimate partner violence participant encountered during the current pregnancy.

Category of IPV	Frequency	Percent
**Physical violence**		
Slapping/throwing something	76	12.4
Pushing/shoving /pulling hair	62	10.1
Hitting with his fist or something that could hurt	73	11.9
Kicking, dragging or beating up	60	9.8
Choking or burning on purpose	20	3.3
Threatening to use or actually using a gun, knife or other weapon	13	2.1
**Overall physical violence**	124	20.3
**Sexual violence**		
Forced to have sexual intercourse you did not want due to fear of you intimate partner	190	31
Forced to have sexual intercourse without your willing	157	25.7
Forced to do something sexual that is degrading or humiliating	73	11.9
**Overall sexual violence**	222	36.3
**Psychological/emotional violence**		
Insulted you or made you feel bad about yourself	154	25.2
Belittled or humiliated you in front of others	98	16
Done things to scare you or intimidate you	120	19.6
Threatened to hurt you or someone you care	39	6.4
**Overall psychological/emotional violence**	202	33.0
**Controlling behavior related violence**		
Tried to keep you from seeing your friends	67	10.9
Tried to restrict contact with family of birth	43	7
Insisted on knowing where you are all times	80	13.1
Get angry if you speak with other man	142	23.2
Suspicious that you are unfaithful	83	13.6
**Overall controlling behavior violence**	186	30.4
**Economic violence**		
Taken your earnings or savings from you against your will	65	10.6
Refused to give you money for household, even when he has money for other things	152	24.8
**Overall economic violence**	165	27.0

**Fig 1:**
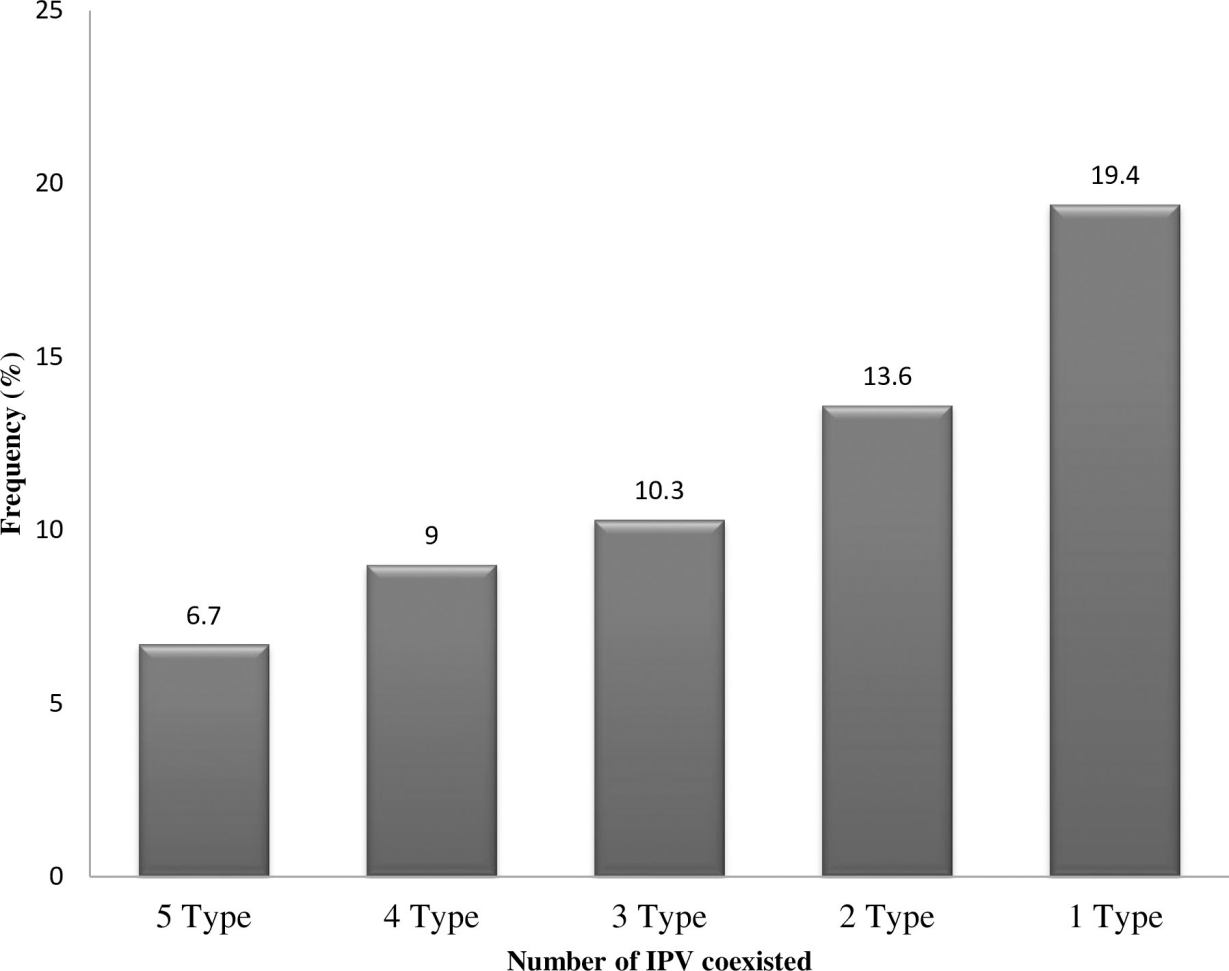
Frequency of IPV coexisted in the pregnant women (n = 612).

Coexistence of different type of IPV was also assessed with among the study participants. Among all study participants, 41 (6.7%) of the pregnant women were the victim the five type of intimate partner violence (physical, sexual, psychological, controlling and economic violence). Four IPV type victim accounts about one in ten of the pregnant women. The three type and two type violence exposed pregnant women were 63 (10.3%) and 83 (13.6%) respectively (**Fig 1**).

### Specific overlap of the common IPV during pregnancy

**Fig 2:**
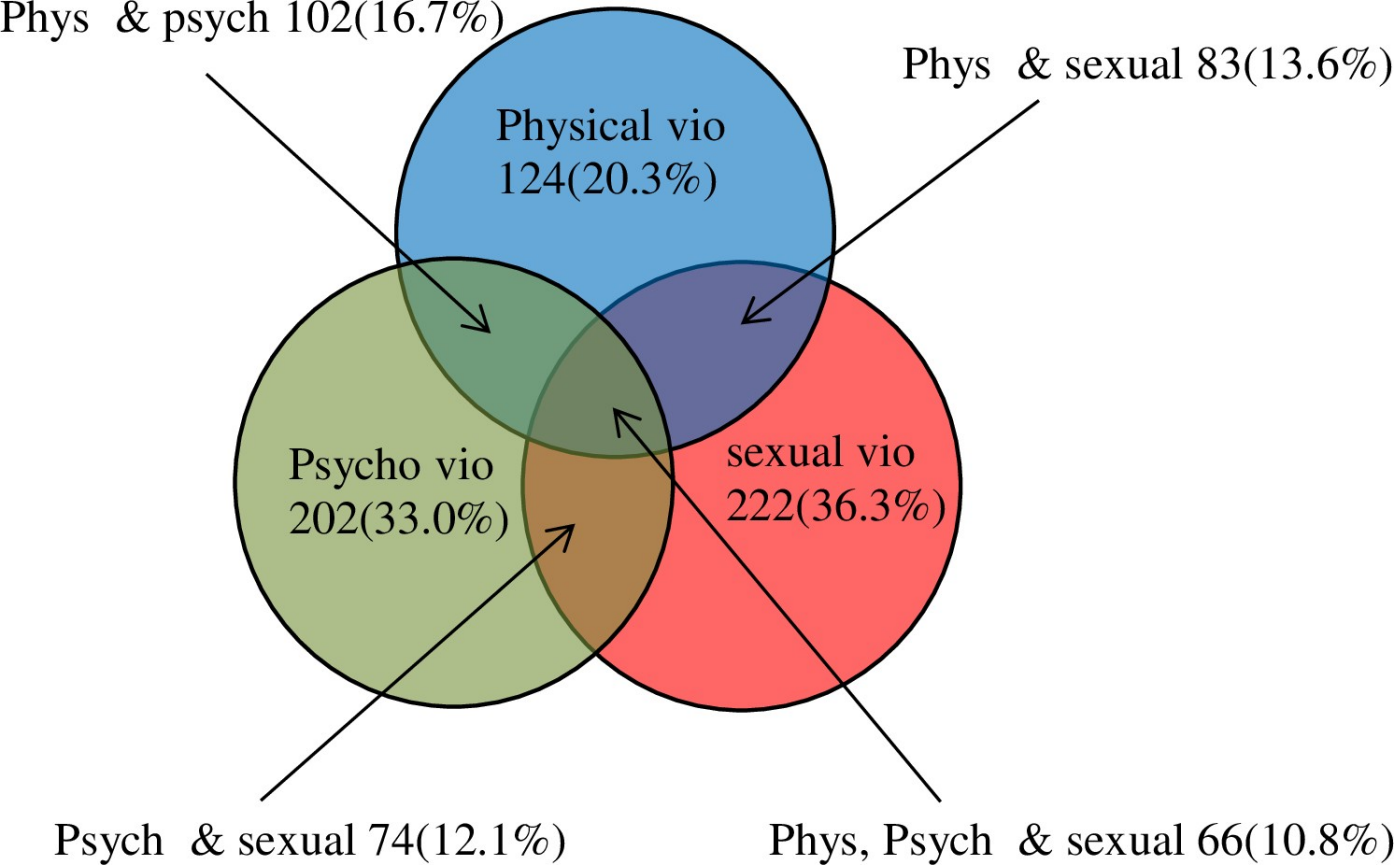
Overlap of the three most common categories of IPV among the pregnant women.

About half (308) of the study participants faced at least one of the three common type violence (physical, sexual and psychological violence) during their current pregnancy. One in ten pregnant women encountered all the three type of the common violence simultaneously. The most frequent coexisted type of IPV was physical and psychological which accounts about 17% among the study participants. The physical and sexual IPV coexistence was reported in 83 (13.6%) of the pregnant women while psychological and sexual was 12.1 **(Fig 2**).

### Factors associated with IPV among pregnant women

Univariable logistic regression analysis was used to screen factors associated with IPV. Socio-demographic variables of the participants and their partner, behavioral characteristics of the participants’ intimate partners, pregnancy and reproductive story of the study participant and other confounders were individually assessed for the presence of association with the IPV. Finally sixteen variables were become eligible for multivariable logistic regression according to the criteria discussed in the method parts.

Among the 16 variables added to multivariable logistic regression, 9 of the variable were not independently associated with IPV. Out of the seven factors associated with intimate partner violence, two of them allied to reproductive history of the pregnant women while the others are linked to their intimate partners’ aspect. Pregnancy intention is one of the factors that independently associated with IPV. Pregnant women who had unwanted pregnancy had about three times more likely encountered IPV than those women who wanted the pregnancy [AOR = 3.3; 95% CI: (1.9–5.5)]. Participants who had a history of miscarriage, abortion and/or still birth had two times odds of exposure to intimate partner violence [AOR = 2.1; 95% CI: (1.2–3.6)] (**Table 4**).

**Table 4:** Factors associated with IPV among the pregnant women.

Variables	IPV exposed	IPV None exposed	COR (95% CI)	AOR (95% CI)	P-Value
Educational status of participants					
Illiterate	184	89	1.89 (1.4–2.6)*	1.48 (09–2.2)	0.062
Literate	177	162	Ref.	Ref.	
Intention of the current pregnancy					
Unwanted	136	25	5.46 (3.4–8.6)*	3.27 (1.9–5.5)	<0.001**
Wanted	225	226	Ref.	Ref.	
History of miscarriage, abortion and/or still birth					
Yes	87	25	2.87 (1.8–4.6)*	2.06 (1.2–3.6)	0.011**
No	274	226	Ref.	Ref.	
Decision making power					
Husband	162	58	1.63 (0.6–4.3)	0.57 (0.2–1.7)	0.319
Together	187	186	0.58 (0.2–1.5)	1.13 (0.4–3.5)	0.832
Wife and other	12	7	Ref.	Ref.	
Polygyny husband					
Yes	92	26	2.96 (1.8–4.7)*	1.6 (0.9–2.8)	0.095
No	269	225	Ref.	Ref.	
Age of intimate partner					
18–29	50	84	Ref.	Ref.	
≥30	311	167	3.12 (2.1–4.6)*	1.85 (1.2–2.9)	0.010**
Husband ever fight with other person					
Yes	150	32	4.86 (3.2–7.4)*	2.81 (1.7–4.6)	<0.001**
No	211	219	Ref.	Ref.	
Alcohol drinking habit					
Yes	79	19	3.42 (2.0–5.8)*	2.86 (1.5–5.4)	0.001**
No	282	232	Ref.	Ref.	
Khat chewing habit					
Yes	171	62	2.74 (1.9–3.9)*	1.66 (1.1–2.6)	0.022**
No	190	189	Ref.	Ref.	
Smoking habit					
Yes	121	16	7.40 (4.2–12.8)*	2.65 (1.4–4.9)	0.002**
No	240	235	Ref.	Ref.	

* -significant in univariable analysis

**-significant in the multivariable analysis

Substance using habit and behavioral factors of pregnant women’s intimate partners were the other factors independently associated with IPV. Pregnant women who had a husband who were drank alcohol [AOR = 2.9; 95% CI: (1.5–5.4)], who were chewed Khat [AOR = 1.7; 95% CI: (1.1–2.6)] and who were smoked cigarette [AOR = 2.6; 95% CI: (1.4–4.9)] had three times, two times and 2.6 times more chance of encountering IPV, respectively. The pregnant women were asked for behavior of their partner and those participants who had partner who ever fought with other persons encountered IPV three times more than the others [AOR = 2.8; 95% CI: (1.7–4.6)]. Age of participants’ intimate partner was also another factor that independently associated with the violence. Pregnant women who had a husband aged 30 years and older had about two times more chance of facing intimate partner violence [AOR = 1.8; 95% CI: (1.2–2.9)] (**Table 4**).

## Discussions

The severity and the type of the intimate partner violence in pregnancy may determine the severity of the outcome [17, 18]. In the current study 59% [95% CI: (55.0–62.8)] of pregnant women encountered at list one type of IPV. This finding supports prior work in IPV prevalence which reflected 61.8% in Gambia and 55% in South Africa [1, 19]. This finding is higher than studies conducted in other region of Ethiopia [20, 21]. The observed difference may be due to variation in the type of violence included in the study. Most of the studies only focused on physical violence while in our case the five type of the intimate partner violence were include. In our study, about 6.7% of the pregnant women faced all the five types of violence while more than one in ten women encountered the three common type of violence (physical, sexual and psychological). Although such high prevalence of IPV was observed in the low income country, required attention is not yet given for violence during the pregnancy [10].

Sexual violence was frequent type of violence which encountered by more than one third of the pregnant women followed by psychological/emotional violence. This finding is higher than the studies conducted in Brazil [18], Hadiya zone [20]and Tanzania [22].Physical violence was less frequent type of violence which accounts for about one fifth of the study participants. Our finding of physical violence was in line with the study conducted in Tigray region [21]. In contrast, the current result is quite higher than studies conducted in Brazil which was 3% [18], and study conducted in Ethiopia Yirgalem town [23] and Hadiya zone [20]. This difference may be observed due to lack of awareness, low level of education, society perception on IPV in our study area.

Substance using habit was one of the factors that were independently associated with IPV among pregnant women in this study. Pregnant women with partners who drank alcohol were more likely to be the victim of IPV. This is consistent with other findings conducted in Ethiopia [20, 21] and other African countries [13, 15, 24]. Alcohol drinking raises levels of aggression, misunderstanding of verbal or non-verbal cues, increased risk taking behavior and alcohol usage might be a source of dispute in relationships [25].

Pregnant women who had smoker and Khat chewer husband were more likely exposed to IPV. Women with Khat chewer husband had about two times more chance encountering IPV. Khat is stimulant plant that abuses a person who frequently used. Smokers and Khat chewer emotion fluctuates based on their smoking pattern and this can influence the relationship between partners. Study conducted in Gambia also reported that smoking cigarette associated with IPV [1]. A review of literature on IPV in Ethiopia showed that those women who had husband with Khat chewing habit were victim of IPV [9]. In our study, an intimate partner with behavior of fighting with other men was also strongly associated with IPV of pregnant women. Other studies also reported that intimate partner who involved in fighting with someone were more likely to be violent against their pregnant wife’s [15, 25].

Older (30 year old and above) intimate partners were twice more likely to be violent to their pregnant women than their younger ones. Similarly, the study in North West Ethiopia reported that as the age of husbands goes older the occurrence of domestic violence increased [10]. The age discrepancy between women and their partners could be the possible reason for the increased odds of violence among oldest intimate partners. In our study, there was a big difference between the age of the pregnant women and their intimate partners. The mean age for women was 26.3 ± 5.8 while for their male partner it was 36.4 ±9.8. Nearly 90% of the pregnant women were in the age group less than 35 years while their intimate partners with related age were about one fifth. The age difference between the partners might affect communication and understanding that lead to violation. Study conducted in Tanzania also reported that young age pregnant women were more violated by their intimate partners who were of older age [22].

The intention of pregnancy was strongly associated with IPV of the study participant. Similarly, other studies reported more violence in women who had an unintended pregnancy [15, 26]. In fact it is difficult to identify which one came first; the violence or lack of intention for the pregnancy. Sometimes lack of interest for the pregnancy might appear after the women faced the violence. Identification of this may need another farther study. Pregnant women who had a history of miscarriage, abortion and/or still birth had two times more chance of exposure to intimate partner violence. The association between adverse birth outcome and IPV is an evidence for the effect of violence on the child health. Other studies also showed that intimate partner violence had an effect on maternal and child health [8, 17, 18].

Limitations related to cross-sectional design and institutional-based studies are the potential limitations of this study. We have included those pregnant women who came for ANC irrespective of their gestational age and this might have underestimated the prevalence of IPV. Fortunately, more than half of our study participants were in the third trimester of pregnancy. Variation of type of violence was observed among previously conducted studies and made comparison difficult for some type of violence. To minimize this problem in our study we had included all type of violence that may have been observed during pregnancy and we had calculated the prevalence separately for all forms of IPV and the overlap of the violence was indicated. Recall bias and social desirability bias may be the other potential limitation of the study. To minimize recall bias the study only focused on the current pregnancy and female data collectors and supervisors were used to minimize social desirability bias.

## Conclusion

The prevalence of intimate partner violence in the Bale zone is high as compared to other areas. Six in ten women experienced at least one act of IPV during pregnancy. Sexual and psychological violence were the most frequent type of IPV. Alcohol drinking habit, Khat chewing, cigarette smoking, fighting with other men and older age were pregnant women’s intimate partner related factors that were independently associated with the IPV. Pregnancy and reproductive history such as miscarriage, abortion and/or still birth and unwanted pregnancy were also significantly associated with IPV against pregnant women. IPV prevention should be incorporated in high school curriculum as well as sexual and reproductive health education programs. Creating the community awareness to change beliefs that are culturally embedded in collaboration with the indigenous leadership, community leader, and other key stakeholders is mandatory. Policy makers need to consider screening for IPV in the antenatal care service as a component. It is also better to include IPV as one component of community health extension package. Those identified behavioral factors call for policy interventions. Further studies that show the effect of intimate partner violence on the pregnancy outcome need to be conducted.

## Supporting information

S1 Questionnaire Questionnaire. (DOCX)

S1 Data Data used in the study. (SAV)
