# Correction: Intimate partner violence and its associated factors among pregnant women in Bale Zone, Southeast Ethiopia: A cross-sectional study

**DOI:** 10.1371/journal.pone.0221442

**Published:** 2019-08-15

**Authors:** Bikila Lencha, Gemechu Ameya, Girma Beressa, Zanebe Minda, Gemechu Ganfure

The third author's name is spelled incorrectly. The correct name is: Girma Beressa. The correct citation is: Lencha B, Ameya G, Beressa G, Minda Z, Ganfure G (2019) Intimate partner violence and its associated factors among pregnant women in Bale Zone, Southeast Ethiopia: A cross-sectional study. PLoS ONE 14(5): e0214962. https://doi.org/10.1371/journal.pone.0214962

## References

[1] Lencha B, Ameya G, Baresa G, Minda Z, Ganfure G (2019) Intimate partner violence and its associated factors among pregnant women in Bale Zone, Southeast Ethiopia: A cross-sectional study. PLoS ONE 14(5): e0214962 10.1371/journal.pone.0214962 31042713 PMC6494036

