# Supplementary material for: Intimate partner violence and its associated factors among pregnant women in Bale Zone, Southeast Ethiopia: A cross-sectional study
Source: PLoS One. 2019 May 1;14(5):e0214962. doi: 10.1371/journal.pone.0214962 (PMC6494036; doi:10.1371/journal.pone.0214962)
Supplement: S1 Questionnaire — (DOCX) [file pone.0214962.s001.docx]

# Individual consent form

Individual consent form to assess Intimate Partner Violence against pregnant women and associated factors among pregnant women in Bale Zone South East Ethiopia

Hello, my name is _______________. I work for Madda Walabu University. We are conducting a survey in Bale Zone to learn about Intimate Partner Violence against pregnant women. You have been chosen by chance to participate in the study. I want to assure you that all of your answers will be kept strictly secret. I will not keep a record of your name or address. You have the right to stop the interview at any time, or to skip any questions that you don’t want to answer. There is no right or wrong answers. Some of the topics may be difficult to discuss, but many women have found it useful to have the opportunity to talk. Your participation is completely voluntary but your experiences could be very helpful to other women in Ethiopia. Do you have any questions? (The interview takes approximately 30 minutes to complete). Do you agree to be interviewed? **Note whether respondent agrees to interview.**

[ ] Does not agree to be interviewed. Thank participant for her time and end interaction.

[ ] Agrees to be interviewed. Is now a good time to talk?

It’s very important that we talk in private. Is this a good place to hold the interview, or is there somewhere else that you would like to go?

I certify that above consent has been read by interviewer and I have a willing to participate in the study.

Signed: ______________________________________ Date: ______________________

# Questionnaire

Questionnaire to assess intimate partner violence and its associated factors among pregnant women in Bale Zone Southeast Ethiopia

| 01 | Code of Questionnaire |  |
| --- | --- | --- |
| 02 | Woreda |  |
| 03 | District name |  |
| 04 | Name of Health institute |  |

## Section I

**Socio-demographic characteristics of the pregnant women and her intimate partner**

| **S. No** | **Questions** | **Response** |  | | **Skip** |
| --- | --- | --- | --- | --- | --- |
| **101** | Your Age in full years | **[______]**in years |  | |  |
| **102** | What is your Religion? | 1. Muslim 2. Orthodox 3. Protestant 4. Others specify_______ | | |  |
| **104** | What is your current  Marital relationship | 1.Married and lived with partner  2.Have regular partner but living apart | | |  |
| **103** | Ethnicity | 1. Oromo 2. Amhara 3. Tigre 4. Others specify______ | | |  |
| **104** | Occupation | 1. Farmer 2. Employer 3. Merchant 4. Housewife 5. Others specify______ | | |  |
| **105** | Educational status | 1. Unable to read and write 2. Able to read and write 3. Primary(1-8) 4. Secondary (9-12) 5. Tertiary (diploma and above) | | |  |
| **106** | Monthly income | ____________ (in **ETB**) | |  |  |
| **107** | Residence | 1. Urban 2. Rural | | |  |
| **108** | With whom do you live? | 1. With your husband 2. With husband family 3. With your family 4. Others ________ | | |  |
| **109** | Family number | ________(in number) | |  |  |
| **200** | Who will make decision on household issue | 1. Husband 2. Wife 3. Jointly | |  |  |
| **201** | Which of the following does your household have? more than one  answer is possible |  | |  |  |
|  | 1. Electricity? 2. Wrist watch? 3. Radio? 4. Television? 5. Mobile telephone? 6. Fixed telephone? 7. Refrigerator 8. Sofa (seat) 9. Electric-mitad 10. Kerosene? 11. Bicycle? 12. motorcycle 13. Horse cart? 14. Car or truck? 15. Jewelry (gold, silver) | 1. 1. Yes 2. No 2. 1. Yes 2. No 3. 1. Yes 2. No 4. 1. Yes 2. No 5. 1. Yes 2. No 6. 1. Yes 2. No 7. 1. Yes 2. No 8. 1. Yes 2. No 9. 1. Yes 2. No 10. 1. Yes 2. No 11. 1. Yes 2. No 12. 1. Yes 2. No 13. 1. Yes 2. No 14. 1. Yes 2. No 15. 1. Yes 2. No 16. 1. Yes 2. No | | |  |
| **202** | Age of your intimate partner | **________________**in years | | |  |
| **203** | Occupation your intimate partner | 1. Farmer 2. Employer 3. Merchant 4. Others specify______ | | |  |
| **204** | Educational status of your intimate partner | 1. Unable to read andwrite 2. Able to read and write 3. Primary(1-8) 4. Secondary (9-12) 5. Tertiary (diploma and above) | | |  |
| **205** | Does your intimate partner drink alcohol | 1. Yes 2. No 3. I don’t know | | |  |
| **206** | Does your intimate partner chew chat? | 1. Yes 2. No 3. I don’t know | | |  |
| **207** | Does your intimate partner smoke cigarette? | 1. Yes 2. No 3. I don’t know | | |  |
| **208** | Does your intimate partner ever fight (physically aggressive) with others men? | 1. Yes 2. No 3. I don’t know | | |  |
| **209** | Have you ever tested for HIV | 1. Yes 2. No | | | If No skip to section II |
| **300** | If yes to Q **209, what was the result?** | 1. Positive 2. Negative 3. I don’t know | | |  |

## Section II

**Types of intimate partner violence**

When two people marry or live together, they usually share both good and bad moments. I would now like to ask you some about your situation after having the current pregnancy and how your husband/intimate partner was treating you. I would again like to assure you that your answers will be kept secret.

| **S. No** | **Question** | **Response** |  | **Skip** |
| --- | --- | --- | --- | --- |
| **301** | During the current pregnancy, did your partner/husband? | **In current pregnancy** | Does this happened in the **past 12 months** before current pregnancy |  |
|  | A) Slapped you or thrown something at you that could hurt you? | 1. Yes  2. No | 1. Yes  2. No |  |
|  | b) Pushed you or shoved you or pulled your hair? | 1. Yes  2. No | 1. Yes  2. No |  |
|  | c) Hit you with his fist or with something else that could hurt you? | 1. Yes  2. No | 1. Yes  2. No |  |
|  | d) Kicked you, dragged you or beat you up? | 1. Yes  2. No | 1. Yes  2. No |  |
|  | e) Choked or burnt you on purpose? | 1. Yes  2. No | 1. Yes  2. No |  |
|  | f) Threatened to use or actually used a gun, knife or other weapon against you? | 1. Yes  2. No | 1. Yes  2. No |  |
|  | **VERIFY WHETHER ANSWERED YES TO ANY QUESTION ON PHYSICAL VIOLENCE SEE QUESTION 301 (A –F)** | 1. **Yes** 2. **No** | 1. **Yes** 2. **No** |  |
| **302** | During the current pregnancy, did your partner/husband? | **In current pregnancy** | Does this happened in the **past 12 months** before current pregnancy |  |
|  | A) Forced you to have sexual intercourse without you interest? | 1. Yes  2. No | 1. Yes  2. No |  |
|  | B) Did you ever have sexual intercourse you did not want to because you were afraid of what your partner might do? | 1. Yes  2. No | 1. Yes  2. No |  |
|  | C) Did your partner or any other partner ever forced you to do something sexual that you found degrading or humiliating? | 1. Yes  2. No | 1. Yes  2. No |  |
|  | **VERIFY WHETHER ANSWERED YES TO ANY QUESTION ON SEXUAL VIOLENCE SEE QUESTION 302 (A –C)** | **1. Yes**  **2. No** | **1. Yes**  **2. No** |  |
| **303** | Psychological violence (4 Questions) | **In current pregnancy** | Does this happened in the **past 12 months** before current pregnancy |  |
|  | A) Has your partner ever; insulted you or made you feel bad about yourself? | 1. Yes  2. No | 1. Yes  2. No |  |
|  | B) Belittled or humiliated you in front of other people? | 1. Yes  2. No | 1. Yes  2. No |  |
|  | C) Done things to scare or intimidate you on purpose (e.g., by the way he looked at you, by yelling and smashing things)? | 1. Yes  2. No | 1. Yes  2. No |  |
|  | D) Threatened to hurt you or someone you care about? | 1. Yes  2. No | 1. Yes  2. No |  |
|  | **VERIFY WHETHER ANSWERED YES TO ANY QUESTION ON PSYCHOLOGICAL VIOLENCE SEE QUESTION 303 (A –D)** | **1.Yes**  **2. No** | **1. Yes**  **2. No** |  |
| **304** | Controlling behavior (5 questions) | **In current pregnancy** | Does this happened in the **past 12 months** before current pregnancy |  |
|  | A) Tried to keep you from seeing your friends? | 1. Yes  2. No | 1. Yes 2. No |  |
|  | B) Tried to restrict contact with your family of birth? | 1. Yes  2. No | 1. Yes 2. No |  |
|  | C) Insisted on knowing where you are all times? | 1. Yes  2. No | 1. Yes 2. No |  |
|  | D) Acted jealous and get angry if you speak with another man? | 1. Yes  2. No | 1. Yes 2. No |  |
|  | E) Often been suspicious that you are unfaithful? | 1. Yes  2. No | 1. Yes 2. No |  |
|  | **VERIFY WHETHER ANSWERED YES TO ANY QUESTION ON CONTROLLING BEHAVIOR VIOLENCE SEE QUESTION 304 (A –E)** | **1.Yes**  **2. No** | **1. Yes**  **2. No** |  |
| **305** | Economic violence (two questions): | **In current pregnancy** | Does this happened in the **past 12 months** before current pregnancy |  |
|  | A) Has your partner ever; taken your earnings or savings from you against your will? | 1. Yes  2. No | 1. Yes  2. No |  |
|  | B) Refused to give you money for household expenses, even when he has money for other things? | 1. Yes  2. No | 1. Yes  2.No |  |
|  | **VERIFY WHETHER ANSWERED YES TO ANY QUESTION ON ECONOMIC VIOLENCE SEE QUESTION 305 (A & B)** | **1.Yes**  **2. No** | **1. Yes**  **2. No** |  |
| **306** | Have you witnessed violence between parents as a child? | 1. Yes  2. No | |  |

## Section III

**Pregnancy and reproductive history of the pregnant women**

| **No** | **Question** | **Response** | **skip** |
| --- | --- | --- | --- |
| **400** | Now I would like to ask about all the births you have had during your life. Have you ever given birth before? | 1.Yes  2. No | If no skip to No.**402** |
| **401** | How many sons to whom you given birth are alive now? | **__________** |  |
| **402** | When you get pregnant for the current pregnancy, did you want to get pregnant at that time? | Yes  No |  |
| **403** | How many months pregnant are you? (or check from registration)  RECORD NUMBER OF COMPLETED MONTHS | **________months** |  |
| **404** | Have you ever had a pregnancy that miscarried, was aborted or ended in stillbirth? | 1.Yes  2. No |  |
| **405** | Are you attending antenatal care for the current pregnancy at health facility? | 1.Yes  2.No |  |
| **406** | What is the sequence of your marriage to your husband? | 1. First 2. Second 3. Third and above |  |
| **407** | What kind marriage ceremony was undertaken when you married your current husband? | 1. Religious 2. Customary 3. No ceremony 4. Other ____ |  |
| **408** | Who choose your husband? | 1. I myself 2. Both of us 3. My family 4. His family 5. Other_____ |  |
| **409** | Did you married him voluntarily? | 1. Yes 2. No |  |
| **500** | Was there dowry/ bride payment during the marriage? | 1. Yes 2. No 3. I don’t know 4. Other______ |  |

## Section IV

**Only for pregnant women any form of the above mentioned Violence**

| **No** | **Question** | **Response** |
| --- | --- | --- |
| 600 | What was your response to violent behavior of your intimate partner | 1. nothing 2. defend 3. reported to local police 4. seek health care 5. separation 6. involving family 7. others_____________ |
| 601 | If your response to Q 600 is ‘nothing’ why? | 1. He prevented me support 2. Children 3. Others ________________ |
| 602 | Did you get modern health care | 1. Yes 2. No |
| 603 | Do you think it is right for the husband to beat his wife | 1. Yes 2. No |
